# CD34 + tumours of the orbit including solitary fibrous tumours: a six-case series

**DOI:** 10.1186/s12886-017-0455-x

**Published:** 2017-04-27

**Authors:** Su Kyung Jung, Ji Sun Paik, Gyeong Sin Park, Suk-Woo Yang

**Affiliations:** 1 Eye clinic, Hospital, National Cancer center, Gyeonggi-do, Korea; 2 Department of Ophthalmology and Visual Science, College of Medicine, Seoul St. Mary’s Hospital, The Catholic University of Korea, 505 Banpo-dong, Seocho-gu, Seoul, 137–701 Korea; 3 Department of Pathology, College of Medicine, Seoul St. Mary’s Hospital, The Catholic University of Korea, Seoul, Korea

**Keywords:** CD34, Solitary fibrous tumour, Fibrous histiocytoma, Haemangiopericytoma, Mesenchymal tumour

## Abstract

**Background:**

To report six cases of CD34+ fibroblastic mesenchymal tumours, which are uncommon neoplasms in the orbit.

**Case presentation:**

Six patients presenting with proptosis and palpable mass who were later diagnosed with fibrous solitary tumours, fibrous histocytoma or haemangiopericytoma in the orbit were included. All patients received radiologic examinations and surgical excision for histopathology and immunohistochemistry examinations. Five patients had no recurrence after a minimum follow-up of 12 months. One patient (case 6) experienced recurrence twice, and had debulking surgeries each time. At present, the patient still has remnant tumour in the orbit, but no growth has been detected during the past two years. The tumour size will be closely monitored.

**Conclusions:**

Even though fibroblastic tumours are rarely found in the orbit, they can present as a palpable mass with proptosis. Complete surgical excision is important for long-term prognosis, and immunohistochemical study is helpful for confirming pathologic diagnosis.

## Background

CD34+ fibroblastic tumours of the orbit are uncommon neoplasms that have not been fully explored. These tumours originate from the mesenchyme and are consistently immunoreactive for CD34 with benign to uncertain behaviour. Haemangiopericytoma, solitary fibrous tumour, giant cell angiofibroma, and fibrous histiocytoma are all CD34+ fibroblastic tumours with similar features [1].

In this report, we describe six cases of CD34+ fibroblastic tumours found in the orbit and conduct a review of the literature discussing the clinical, histopathological and immunohistochemical features of the tumours found to date.

## Case presentation

Table 1 summarizes the clinical characteristics of our six cases of CD34+ fibroblastic tumours found in the orbit.

**Table 1:** Summarized clinical features for 6 patients with CD34+ tumours of the orbit

Case No.	Gender/Age (years)	Symptoms	Location	Final diagnosis	Treatment	Outcome
1	F/23	Proptosis, chemosis and EOM^a^ limitation	Retrobulbar	Solitary fibrous tumour	Debulking surgery + gamma knife radiosurgery	No recurrence for 2 years
2	F/29	Proptosis	Superomedial extraconal	Solitary fibrous tumour	Complete surgical removal	No recurrence for 2 years
3	M/50	Periorbital swelling	Lower eyelid	Solitary fibrous tumour	Complete surgical removal	No recurrence for 4 years
4	F/15	Proptosis	Retrobulbulbar	Solitary fibrous tumour	Complete surgical removal	No recurrence for 3 years
5	F/54	Palpable mass	Inferomedial extraconal	fibrous histiocytoma	Complete surgical removal	No recurrence for 4 years
6	F/42	Periorbital swelling	Medial extraconal and nasal cavity	Haemangio-pericytoma	Repeated debulking surgery	Twice recurrence -> debulking surgery, Still under intensive follow up due to remnant mass without growth

^a^EOM extraocular muscle

### Case 1

**Fig. 1:**
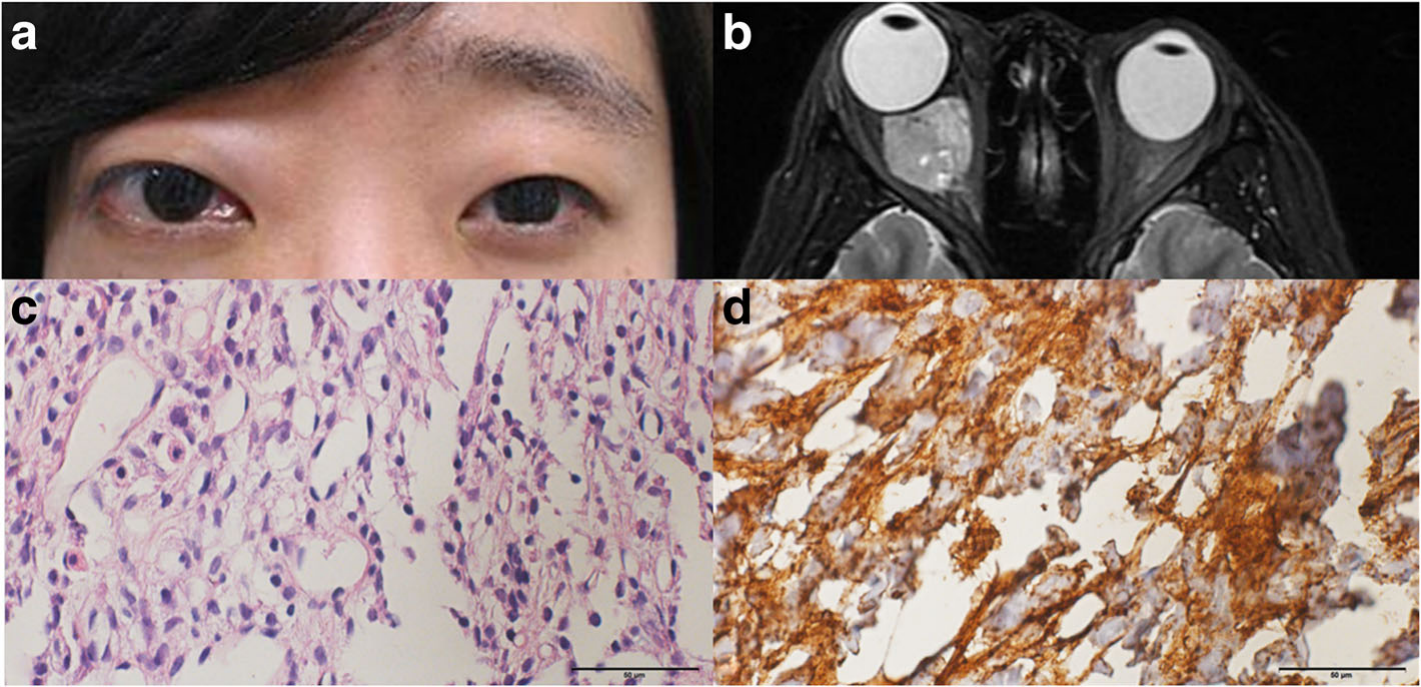
**a** A clinical photograph shows the proptosis and conjunctival chemosis of the right eye. **b** Axial T2-weighted orbital MRI showed a strong enhancing mass at the retrobulbar region of the right orbit. The biopsy specimen showed spindle cells with the myxoid features (H-E stain, x400) **c** and intense reactivity for CD34 (CD34 stain, x 400), **d**

A 23-year-old female presented with a recurrent right proptosis for 12 months. She had a prior history of a right orbital myxoid spindle cell neoplasm removed 3 and 4 years prior in two different hospitals. Despite the operations, the proptosis progressed. Therefore, she was referred to our hospital to receive an additional operation. Ophthalmic examination showed a 6-mm proptosis, inferior conjunctival chemosis of the right eye, and limitation of the extraocular movement in all gazes (Fig. 1a). T2-weighted orbital molecular resonance imaging (MRI) showed a strong enhancing mass in the retrobulbar region of the right orbit of approximately 21 x 20 x 20 mm in size (Fig. 1b). Tumour removal was performed by lateral orbitotomy including the eyelid crease and a temporal area incision without bone removal. The tumour was poorly defined with infiltration into the adjacent tissue, and the visible capsule was not left, so a debulking surgery was performed. After surgery, the patient received gamma knife radiosurgery, and the proptosis in the right eye was reduced to 2 mm. Histological examination of the biopsy specimen showed spindle cells with atypical myxoid features (Fig. 1c). The tumour cells showed intense reactivity for CD34, CD31, CD68, and p53, and no reactivity for CD56a or S-100 (Fig. 1d). The histopathologic and immunophenotypic features were diagnostic of a solitary fibrous tumour.

### Case 2

**Fig. 2:**
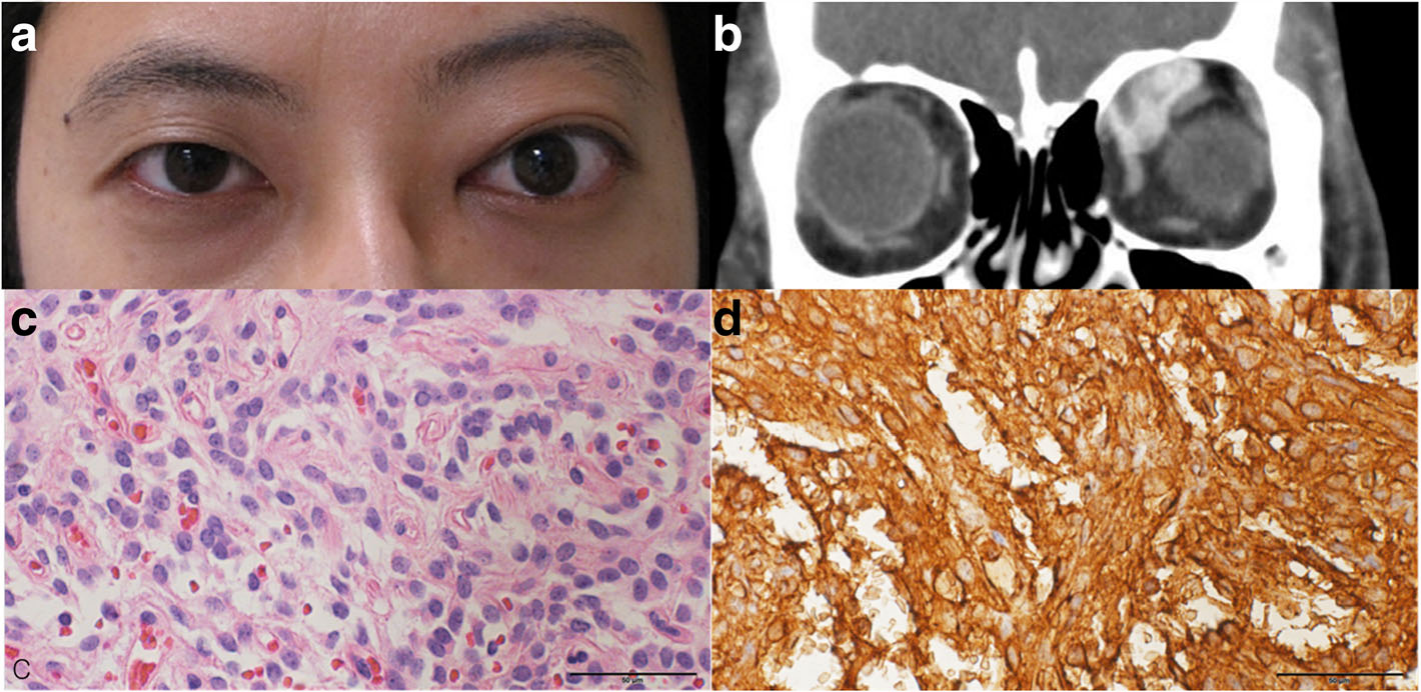
**a** A clinical photograph shows the proptosis of the left eye. **b** Contrast enhanced coronal CT scan shows the heterogeneous enhancing lesion in the left superomedial extraconal space. Histopathologic examination shows spindle cells with atypical vascular structures (H-E stain, x400) **c** and intense reactivity for CD34 (CD34 stain, x 400), **d**

A 29-year-old female presented with a left proptosis for 10 months. Ophthalmic examination showed a 2.5 mm proptosis of the left eye without limitation of extraocular movement (Fig. 2a). The patient had previously been diagnosed with hyperthyroidism and had taken methimazole for seven years. A computed tomography (CT) examination detected an approximately 2.0 cm heterogeneous enhancing lesion without bone erosion in the left superomedial extraconal space (Fig. 2b). The mass was completely excised by anterolateral orbitotomy. After surgery, the proptosis improved. Histopathologic examination showed spindle cells with atypical vascular structures and intense reactivity for CD34 (Fig. 2c, d).

### Case 3

**Fig. 3:**
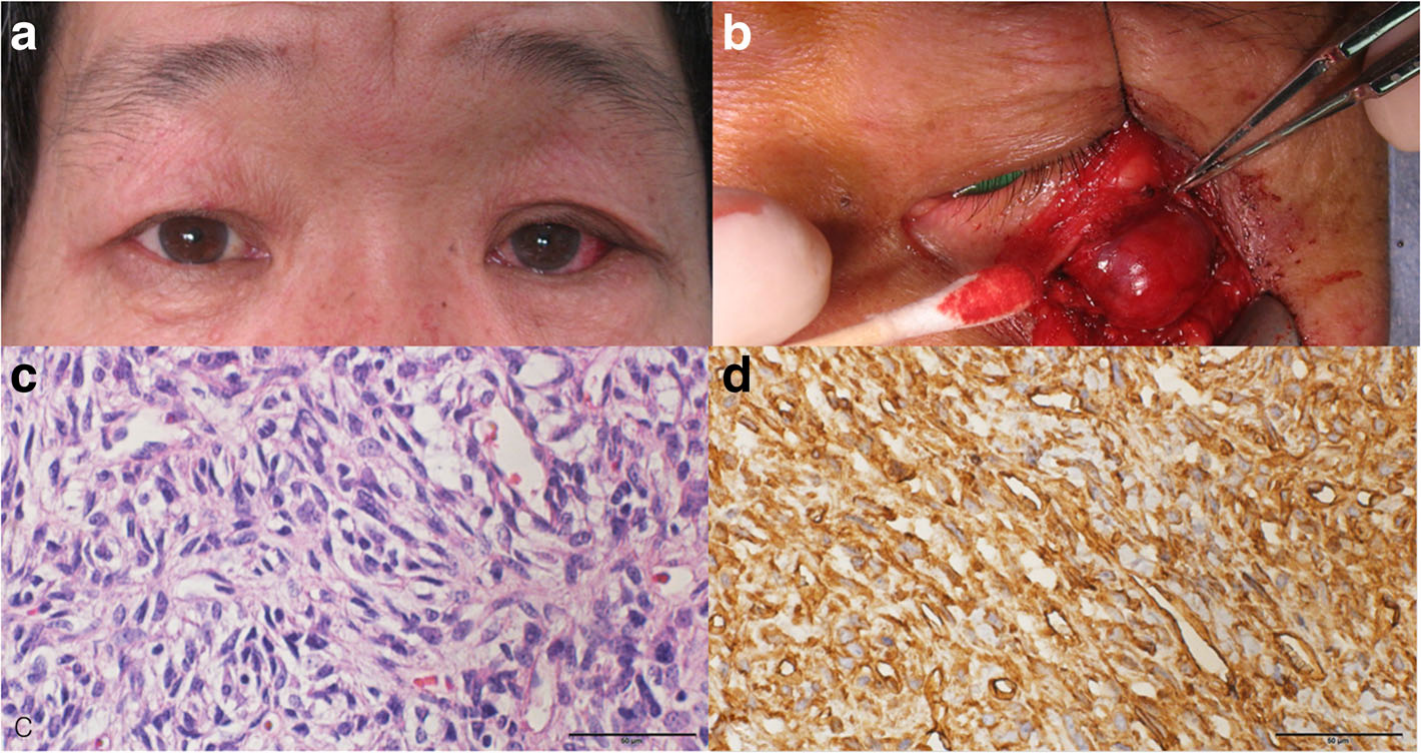
**a** A clinical photograph shows a periorbital swelling with palpable mass in the left eye. **b** The surgically removed mass as a reddish surface and hard elastic consistency. The specimen has spindle-shaped cells (H-E stain, x400) without mitosis or nuclear atypism **c** and strong reactivity for CD34 (CD34 stain, x 400), **d**

A 50-year-old male was referred to our clinic with periorbital swelling that had existed for one year (Fig. 3a). A 4 mm mass was observed in the inferior bulbar conjunctiva of the left eye, but other ophthalmic examinations were unremarkable. The mass was completely removed by anterior orbitotomy (Fig. 3b), and the patient was diagnosed as having a solitary fibrous tumour based on histopathologic examination (Fig. 3c, d).

### Case 4

**Fig. 4:**
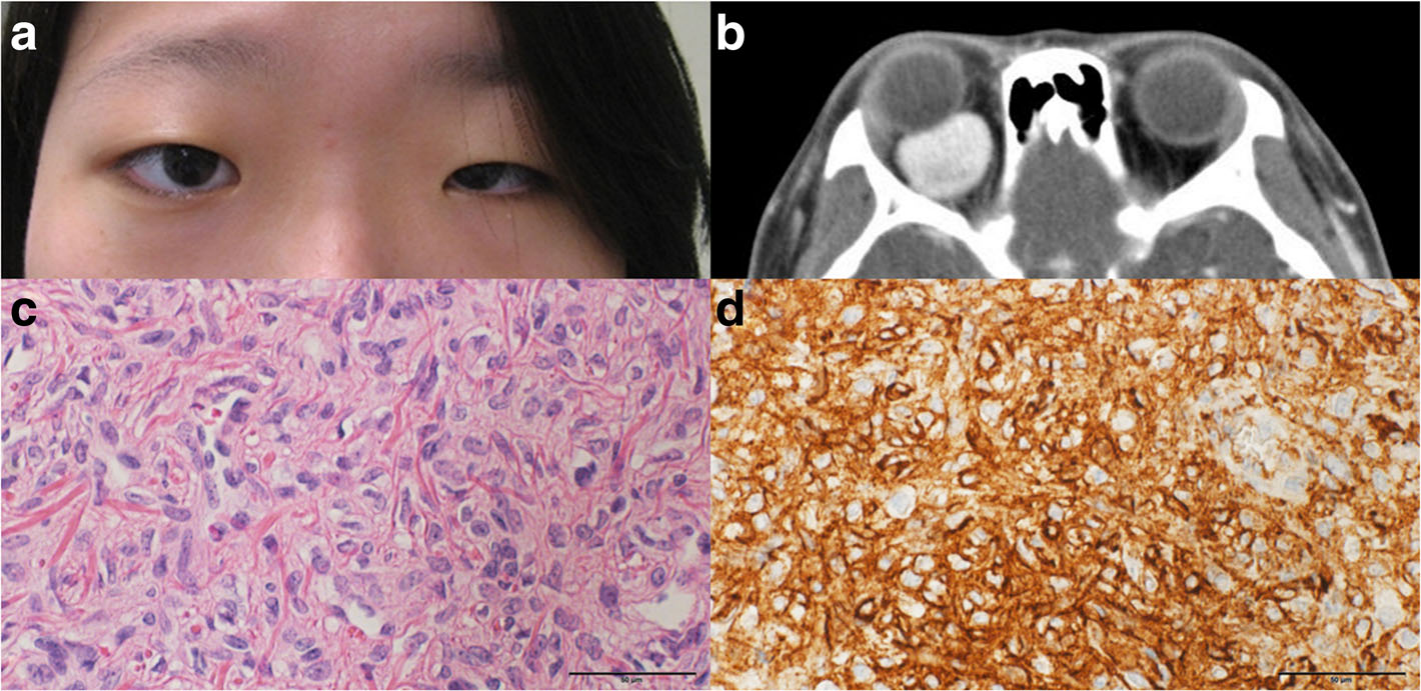
**a** A preoperative photograph shows the proptosis of the right eye. **b** A 2.6 cm enhanced lesion was detected in the right retrobulbar space based on the axial contrast enhanced CT scans. The biopsy specimen shows spindle cells with the eosinophilic cytoplasm (H-E stain, x400) **c** and intense reactivity for CD34 (CD34 stain, x 400), **d**

A 15-year-old female was referred to our clinic with proptosis of the right eye that had existed for several months. Ophthalmic examination showed a 2.5 mm proptosis of the right eye without limitation of extraocular movement (Fig. 4a). CT examination detected a 2.6 cm lesion in the right retrobulbar space (Fig. 4b). The mass was completely excised via a vertical upper eyelid split approach. After surgery, the proptosis improved, and the histopathologic examination showed an extrapleural solitary fibrous tumour of the cellular type and intense reactivity for CD34 (Fig. 4c, d).

### Case 5

**Fig. 5:**
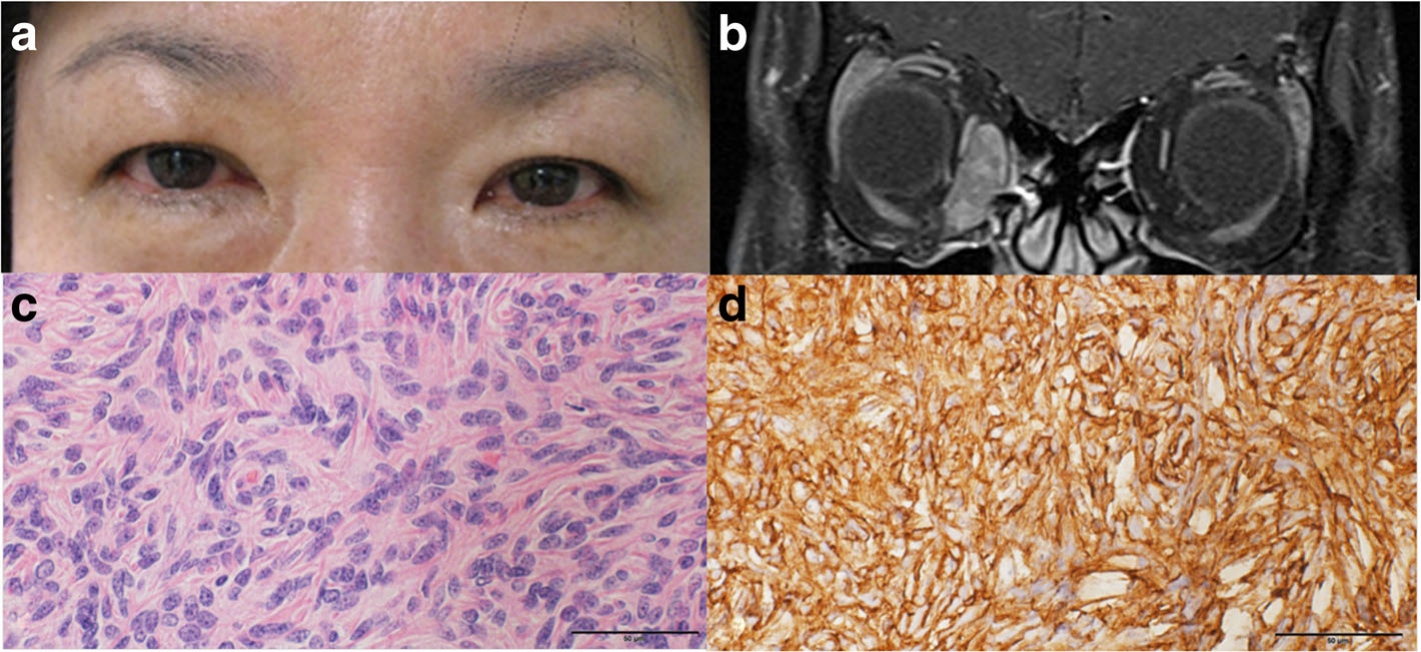
**a** A movable mass is shown in the inferomedial side of the right eye. **b** T1-weighted orbital MRI shows 3 cm mass in the extraconal space of the right eye. The storiform pattern of fibroblasts is shown (H-E stain, x400) **c** and the positive reactivity for CD34 was detected in histopathologic finding (CD34 stain, x 400), **d**

A 54-year-old female was referred to our clinic with a palpable mass in the left lower eyelid that had existed for two months. Ophthalmic examination was unremarkable, except for a movable mass without tenderness (Fig. 5a). T1-weighted orbital MRI showed a 3 cm mass in the right inferomedial extraconal space (Fig. 5b). The mass was completely removed using a subciliary approach. Histopathologic examination revealed a storiform pattern of fibroblasts with mitosis and positive reactivity for CD34, CD68 and vimentin (Fig. 5c, d). The patient was diagnosed as having a fibrous histiocytoma with high proliferation capacity.

### Case 6

**Fig. 6:**
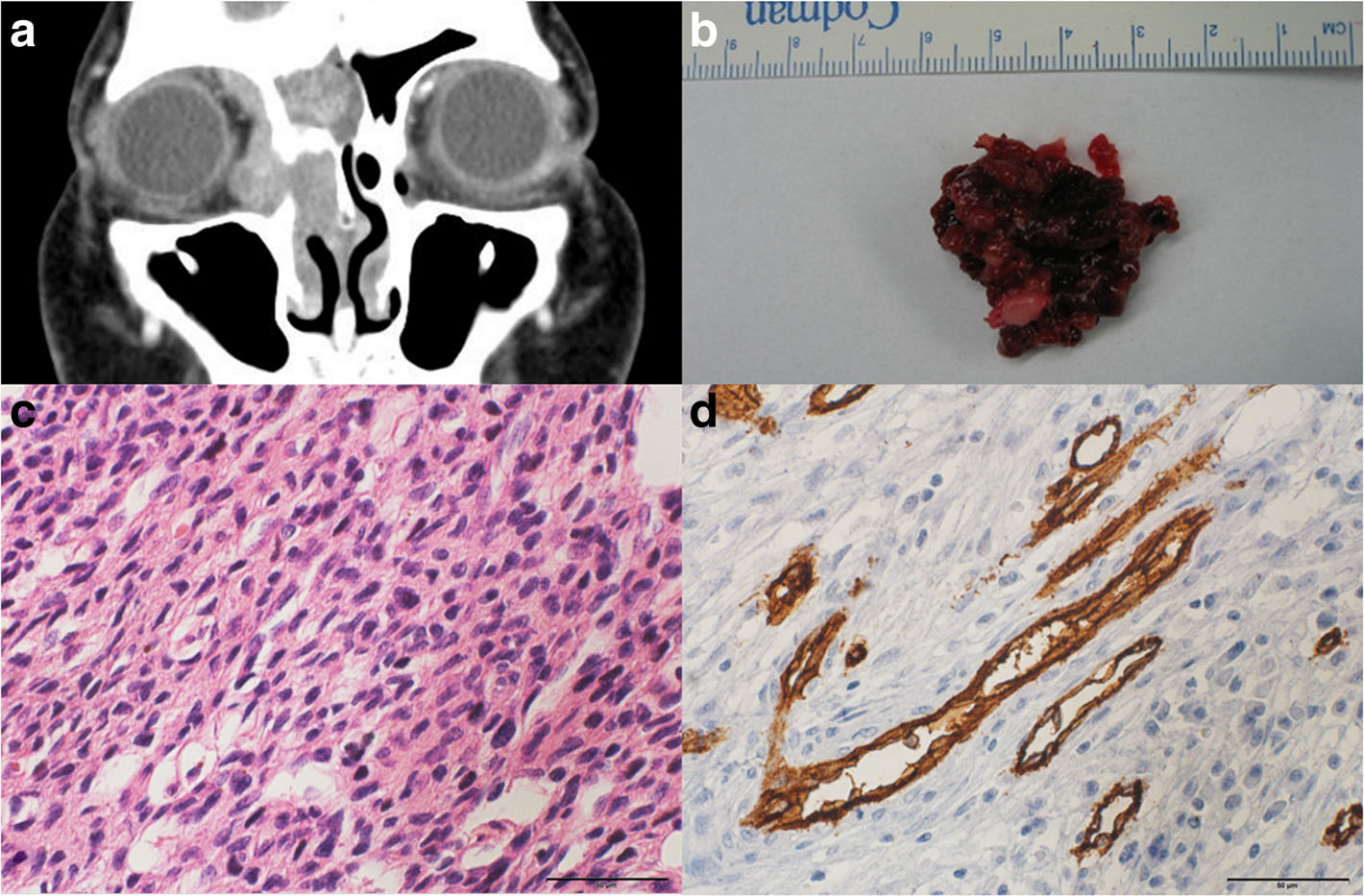
**a** The coronal contrast enhanced orbit CT shows much progression of the mass involving the right ethmoid sinus, frontal sinus, orbit, and some portions of the nasal cavity. **b** The removed tumour has a cystic and fragile structure. The tightly packed spindle-shaped cells (H-E stain, x400) **c** are shown with positive reactivity for CD34 (CD34 stain, x 400), **d**

A 42-year-old female was referred to our clinic with periorbital swelling from otorhinolaryngology. She underwent tumour removal via an endoscopic approach due to glomangiopericytoma in the right nasal cavity that had occurred one year prior. The enhanced orbit CT showed significant progression of the known recurrent mass involving the right ethmoid sinus, frontal sinus, orbit, and some portions of the nasal cavity (Fig. 6a). The tumour was removed using anterior-medial lych incisional orbitotomy and an endoscopic endonasal approach. The tumour was cystic and had a fragile structure (Fig. 6b); therefore, it could not be removed completely. Histopathologic examination revealed tightly packed spindle-shaped cells with positive reactivity for CD34 and vimentin (Fig. 6c, d). The patient experienced recurrence twice and had surgery each time to remove the mass. At present, the patient still has remnant tumour in the orbit, but no growth has been detected. The tumour size will be closely monitored, but no additional surgeries are planned.

## Discussion and Conclusions

In this study, six cases of orbital tumours including fibrous solitary tumours, fibrous histiocytoma and haemangiopericytoma are presented. These tumours can be found anywhere in the body, but they are infrequently found in the orbit. Approximately 80 cases have been reported to date, which exhibit overlapping morphologic and immunophenotypic findings [1, 2]. Therefore, conflicting evidence exists regarding pathologic diagnosis. In this study, we aimed to present the cases and review the current literature, including the histopathologic diagnosis of the tumours.

Solitary fibrous tumours (SFTs) are uncommon tumours described as mesenchymal neoplasms originating in the pleural structures. Although initially described in the pleura, SFTs have subsequently been encountered in extrapleural locations, including the pericardium, mediastinum, peritoneum, nasopharynx, paranasal sinuses, and orbit [3]. The most common ophthalmic manifestation is unilateral proptosis, and it can be accompanied by eyelid swelling, vision disturbances, a palpable mass, tearing or ptosis [4]. On CT and MR imaging, orbital SFT usually presents as a well-defined soft tissue mass with strong enhancement, although bone remodelling is present in cases of suspicious malignant transformation [5]. In pathologic examination, SFT cells are described as spindle-shaped with a small cytoplasm and indistinct nucleoli, and the tumour matrix contains a distinctive thick “ropy” type of collagen between tumour cells [1]. SFTs are consistently, strongly immunoreactive for CD34 (90–100%) [3, 6]. The CD34 protein is a member of a family of single-pass transmembrane sialomucin proteins that are expressed in early haematopoietic and vascular-associated tissue [7]. Therefore, CD34 is expressed early in the cells and embryonic fibroblasts [8]. High reactivity of CD34 is helpful for the diagnosis of SFT, but other tumours that should be differentially diagnosed as an orbital SFT also have variable reactivity.

Haemangiopericytoma (HPC) is also a rare mesenchymal tumour believed to originate from the pericytes of blood vessels [9]. HPC can be found anywhere capillaries are present, but occurrence in the orbit is very rare [9]. Clinically, it presents as a painless slow-growing mass and metastasizes in approximately 12-45% of cases. The common sites of metastases are lung, mediastinum, liver and bone [10]. Histological evaluation of HPC reveals a mixture of spindle-shaped tumour cells with oval nuclei and a small cytoplasm mixed with a network of thin-walled blood vessels or sinusoid-like spaces [2]. HPC cells show high reactivity against vimentin and CD34 but lack immunoreactivity for Epithelial membrane antigen (EMA). These specific immunohistochemical characteristics aid in diagnosis.

Fibrous histiocytoma (FH) is the most common primary mesenchymal neoplasm of the orbit in adults [11]. The upper and nasal portions of the orbit are the most common sites of involvement [11]. FH consists of prominent histiocytes and fibroblasts in a storiform pattern. Immunohistochemical staining consistently reveals that histiocytes are positive for CD68, but some variation in CD34 reactivity exists [1]. In addition, FH is typically classified as benign but exhibits locally aggressive and malignant features based on its histopathologic features, including atypical mitosis and cellular pleomorphism [11]. In addition to histologic differences among the benign, locally aggressive, and malignant tumours, the benign tumours have longer duration of symptoms and smaller size compared with locally aggressive and malignant tumours [11].

As mentioned above, orbital SFT, HPC and FH have overlapping morphologic and immunohistochemical features (Table 2). Haphazardly disposed fibroblast-like cells, indistinct nucleoli, variable stromal collagen, and prominent vasculature with perivascular fibrosis, are known overlapping features. Therefore, some studies have found that light microscopic, ultrastructural, and immunohistochemical analyses reveal few differences between HPC and SFT, leaving considerable overlap between the two neoplasms [12]. In addition, Gengler and Guillou [13] suggested that orbital HPC, FH, giant cell angiofibroma, and SFT represent a spectrum of solitary fibrous tumours from a histopathological point of view. Moreover, the term “haemangiopericytoma” or “cellular variant of solitary fibrous tumour” has frequently been used as the same disease entity in recent publications [2]. In HPC, FH and SFT, debate exists in the current literature despite the general acceptance that they are separate entities. In this study, two patients were diagnosed with orbital SFT, but the pathologic findings showed a rich vascular component that may indicate HPC.

**Table 2:** The morphologic and immunohistochemical feature of orbital solitary fibrous tumour, fibrous histiocytoma and haemangiopericytoma

	Solitary fibrous tumour	Fibrous histiocytoma	Haemangiopericytoma
Histopathology	Spindle shaped cells with scanty eosinophilic cytoplasm	Foamylike histiocytes and spindle-shaped fibroblasts-like cells arranged in a storiform pattern	Staghorn sinusoidal blood vessels with individual tumour cells surrounded by reticulin fibers
CD34	High reactivity	Reactivity?	High reactivity, less diffusely and weaker compared with SFTs
Vimentin	High reactivity	-	High reactivity
S-100	Negative	-	Negative
EMA	Negative	-	Negative
CD68	-	Reactivity	-

The English in this document has been checked by at least two professional editors, both native speakers of English. For a certificate, please see: http://www.textcheck.com/certificate/8raTDB

In addition, we use English editing service from American Journal Experts (http://bit.ly/AJE_BS) during second revision

The prognoses of these fibroblastic tumours are not clear because these tumours are very rare, and only individual cases have been published. Orbital SFTs are generally accepted as benign tumours with a favourable clinical course. However, some cases are aggressive, and cases surrounding tissue invasion have been reported [4]. Therefore, complete surgical resection is critical to diagnose, treat and prevent recurrence and progression. Similarly, complete resection in cases of FH or HPC is important because remnant tumour is associated with local recurrence [11]. In HPC, the histopathological features do not always correlate with biological behaviour, including malignant potential [14]. Therefore, complete surgical removal should be performed regardless of the pathological findings. Occasionally, radiotherapy is used when the lesion is not completely removed [15]. Complete surgical removal was not possible in case 1, a patient with SFT, due to infiltration of the surrounding tissue, or in case 6, a patient with HPC, due to the fragile structure of the tumour. Case 1 did not experience recurrence for 5 years after gamma knife radiosurgery. However, case 6 was not able to receive radiotherapy due to the patient’s personal circumstances. Therefore, the tumour was closely monitored for five years and recurred twice. Additional operations were performed on the recurring tumours, and no additional growth has been observed for the past two years.

In this study, we reported six cases of fibroblastic tumours including SFT, FH and HPC that were consistently immunoreactive for CD34. Controversy exists around the pathologic findings of these tumours because they are highly similar. Performing a complete surgical excision is important for good prognosis, and fibroblastic tumours should occasionally be suspected when a painless, slow-growing orbital mass is detected.

## Abbreviations

HPC: Haemangiopericytoma
FH: Fibrous histiocytoma
SFT: Solitary fibrous tumour

## References

[1] Furusato E, Valenzuela IA, Fanburg-Smith JC, Auerbach A, Furusato B, Cameron JD, Rushing EJ (2011). Orbital solitary fibrous tumor: encompassing terminology for hemangiopericytoma, giant cell angiofibroma, and fibrous histiocytoma of the orbit: reappraisal of 41 cases. Hum Pathol.

[2] Hsu CH, Wei YH, Peng Y, Liao SL (2014). Orbital hemangiopericytoma in an Asian population. J Formos Med Assoc.

[3] Brunnemann RB, Ro JY, Ordonez NG, Mooney J, El-Naggar AK, Ayala AG (1999). Extrapleural solitary fibrous tumor: a clinicopathologic study of 24 cases. Mod Pathol.

[4] Le CP, Jones S, Valenzuela AA (2014). Orbital solitary fibrous tumor: a case series with review of the literature. Orbit.

[5] Kim HJ, Kim HJ, Kim YD, Yim YJ, Kim ST, Jeon P, Kim KH, Byun HS, Song HJ (2008). Solitary fibrous tumor of the orbit: CT and MR imaging findings. Am J Neuroradiol.

[6] Westra WH, Gerald WL, Rosai J (1994). Solitary fibrous tumor. Consistent CD34 immunoreactivity and occurrence in the orbit. Am J Surg Pathol.

[7] Nielsen JS, McNagny KM (2008). Novel functions of the CD34 family. J Cell Sci.

[8] Civin CI, Strauss LC, Brovall C, Fackler MJ, Schwartz JF, Shaper JH (1984). Antigenic analysis of hematopoiesis. III. A hematopoietic progenitor cell surface antigen defined by a monoclonal antibody raised against KG-1a cells. J Immunol.

[9] Stout AP, Murray MR (1942). Hemangiopericytoma: a vascular tumor featuring zimmermann's pericytes. Ann Surg.

[10] Panda A, Dayal Y, Singhal V, Pattnaik NK (1984). Haemangiopericytoma. Br J Ophthalmol.

[11] Font RL, Hidayat AA (1982). Fibrous histiocytoma of the orbit. A clinicopathologic study of 150 cases. Hum Pathol.

[12] Alawi F, Stratton D, Freedman PD (2001). Solitary fibrous tumor of the oral soft tissues: a clinicopathologic and immunohistochemical study of 16 cases. Am J Surg Pathol.

[13] Gengler C, Guillou L (2006). Solitary fibrous tumour and haemangiopericytoma: evolution of a concept. Histopathology.

[14] Croxatto JO, Font RL (1982). Hemangiopericytoma of the orbit: a clinicopathologic study of 30 cases. Hum Pathol.

[15] Setzkorn RK, Lee DJ, Iliff NT, Green WR (1987). Hemangiopericytoma of the orbit treated with conservative surgery and radiotherapy. Arch Ophthalmol.

